# A Case of C5 Vertebral Chordoma in a 73-Year-Old Patient with More Than 8 Years of Follow-Up after Total Piecemeal Spondylectomy

**DOI:** 10.1155/2017/3284131

**Published:** 2017-09-12

**Authors:** Keigo Tanaka, Harutoshi Sakakima, Kazutoshi Hida, Kanako Hatanaka, Kosei Ijiri

**Affiliations:** ^1^Yunomoto Memorial Hospital, Kagoshima, Japan; ^2^School of Health Sciences, Faculty of Medicine, Kagoshima University, Kagoshima, Japan; ^3^Sapporo Azabu Neurosurgical Hospital, Sapporo, Japan; ^4^Department of Pathology, Hokkaido University Hospital, Sapporo, Japan; ^5^Kirishima Orthopedics, Kirishima, Japan

## Abstract

Chordoma arising from the cervical spine is rare and the traditional long-term prognosis is typically poor. Total en bloc spondylectomy with a wide margin is generally accepted to be the most appropriate management for thoracic and lumbar malignant tumors. However, this method is still challenging for the cervical spine because of the proximity of the tumor to the vertebral arteries and neural elements. Here, we report a 73-year-old man with a C5 vertebral chordoma treated with total piecemeal spondylectomy. Histological examination revealed pathognomonic physaliphorous cells with mucus-filled cytoplasm in the tumor, and the ratio of Ki-67-positive cells within the tumor was high (19.0%), showing active proliferation rate. Local recurrences were found at 9 months, 4 years and 2 months, and 6 years after the initial surgery. All the recurrences were encapsulated and isolated and treated with an additional en bloc resection successfully at each stage. Eight years after the initial total piecemeal spondylectomy, the patient maintained his intact neurological status without local recurrence or metastasis. The prognosis of cervical chordoma depends on the patient's age, surgical procedures, and histological features. In this report, we present that piecemeal spondylectomy is an alternative management for aged patients with cervical chordoma, even for those with high MIB-1 index.

## 1. Introduction

Chordoma is a slow-growing, locally invasive, rare malignant tumor restricted to the axial skeleton that occurs with an overall incidence of less than 1 in 1,000,000 people [1–4]. The condition is thought to arise from remnants (rests) of the embryonic notochordal cells [5]. Histological examination revealed that these tumor cells often contain numerous intracytoplasmic vacuoles (physaliphorous) and are separated by variable amounts of intercellular mucin, accounting for 1% to 4% of all malignant bone tumors [5–8]. Men appear to be more susceptible to the development of chordoma than women (ratio 2 : 1), and lesions typically arise in the sacrococcygeal area (29–57%), sphenooccipital area (27–35%), and vertebrae (10–33%) [1, 6, 9, 10]. In the spinal columns, the tumor is located in the cervical spine (15–52%), lumbar spine (33–35%), and thoracic spine (14–17%) [2, 10–12].

Depending on the location, chordoma can involve the nervous system. Compression of the adjacent spinal cord can cause neurological symptoms, such as paralysis, ataxia, loss of proprioception, urinary incontinence, and/or constipation [13]. The location and large size of the tumor at presentation often precluded complete surgical removal in the past, and the long-term prognosis is typically poor [1, 14]. The poor outcome is primarily due to extensive local recurrences and secondary complication [10, 14].

Total en bloc spondylectomy with a wide margin is generally accepted to be the most appropriate management for thoracic and lumbar lesions. However, this method is still technically challenging for the cervical spine because of the proximity of the tumor to the vertebral arteries and neural element. On the other hand, old patients were featured as having significantly longer survival than younger patients with cervical chordoma regardless of the resection methods, either total piecemeal spondylectomy or total en bloc spondylectomy [15].

Here, we report the case of a 73-years-old patient with C5 vertebral chordoma who showed neurological symptoms because of tumor invasion into the neural foramen and canal, compressing the spinal cord and nerve root. We performed total piecemeal spondylectomy and spinal reconstruction. Local recurrences were found at 9 months, 4 years and 2 months, and 6 years after initial surgery. All recurrences were encapsulated and treated with additional resection successfully at each stage. Eight years after the initial total piecemeal spondylectomy, the patient maintains his intact neurological status without local recurrence or metastasis.

## 2. Case Presentation

**Figure 1:**
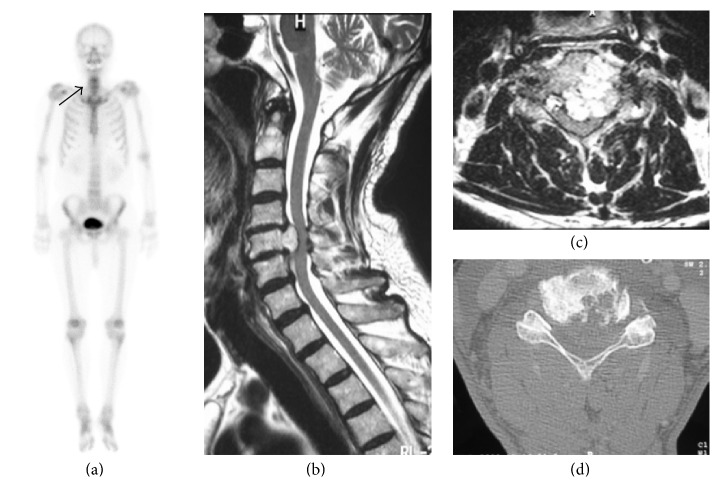
Technetium bone, magnetic resonance imaging (MRI), and computed tomography (CT) scans. (a) Technetium bone scan was useful in localizing the lesion (arrow) and no metastatic lesion was found. (b, c) The MRI scan showed a lesion of increased signal intensity on T2-weighted images in the C5 region, and the tumor is protruding into the spinal canal from the left side of the C5 vertebral body. (d) The CT scan shows the osteolytic lesion in the C5 vertebral body.

**Figure 2:**
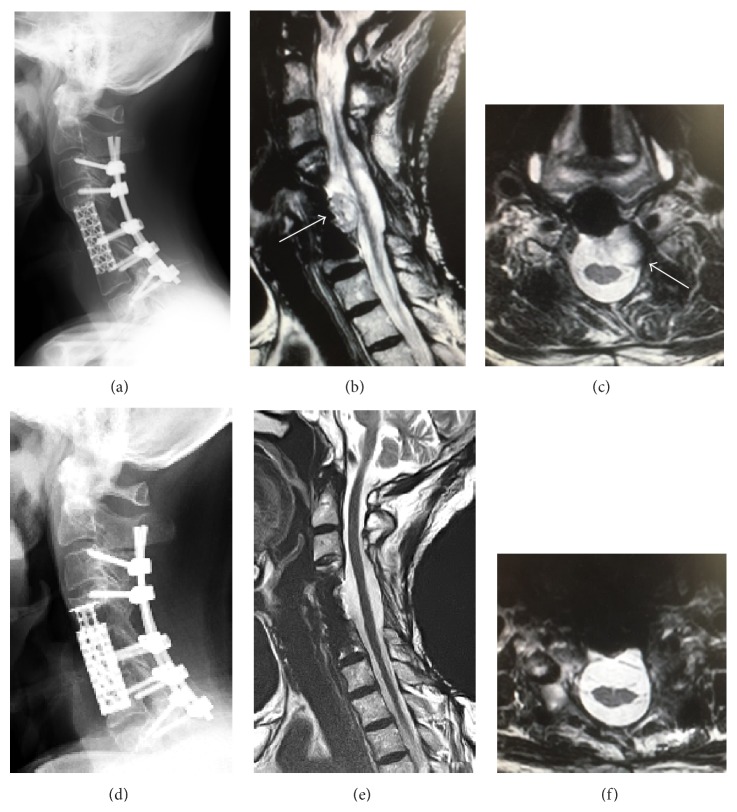
Roentgenogram after surgery. (a) Lateral radiograph showing posterior cervical interbody fusion with the artificial vertebral body of C3 to T1 after the first surgery. (b, c) Sagittal (b) and axial MRI scan (c) at 6 years after the initial surgery with a third recurrence of the tumor. (d) Lateral radiograph of the cervical spine, and sagittal (e) and axial views (f) of the MRI image, showing no recurrence at 8 years 4 months after the first surgery.

The patient was a 73-year-old man who was followed up for left shoulder pain for 1 year in another hospital. Magnetic resonance imaging (MRI) was performed because of progression of signs and symptoms, such as backache and lower limb weakness. Muscle weakness level was 4 in the manual muscle test, with slight difficulty of gait and muscle atrophy of the interossei dorsales muscle bilaterally. The patient complained of a moderate glove-and-stocking-type numbness on the four limbs. The MRI scan showed an area of increased signal intensity on T2-weighted images in the C5 region (Figure 1(b)). At discovery, the tumor was protruding into the spinal canal from the left side of the C5 vertebral body and pressed the dura mater from the front. The neurological symptoms were ascribed to the compression of the spinal cord and nerve roots by an expanding mass partly protruding into the vertebral canal (Figures 1(c) and 1(d)). The metastatic lesion was found on neither the technetium bone scan (Figure 1(a)) nor the positron emission tomography. The patient was treated with surgical resection of the tumor, followed by reconstruction in two stages. As the first stage, a posterior approach was selected for decompression. No heavy ion radiotherapy was performed because the tumor massively invaded into spinal canal and foramen, compressing the spinal cord and nerve root directly. Total laminectomy was performed for C4, C5, and C6. All the posterior elements, including the superior and inferior articular process and pedicles of C5, were resected by using piecemeal spondylectomy with air burr and Kerrison rongeurs. No bony element of C5 was restored without vertebral body, followed by fusion of C3 to T1 with pedicle screws. As the second stage, an anterior approach was performed to remove the total C5 vertebral body, lower bony endplate of the C4 vertebral body with the C4/5 disk, and upper bony endplate of the C6 vertebral body with the C5/6 disk. All these tissues were resected using an air burr and a Kerrison rongeurs. Complete intralesional resection of the tumor was performed by piecemeal, not en bloc resection, followed by spine column reconstruction by using a titanium mesh cage (Figure 2(a)). The patient did not undergo radiotherapy, because the tumor was located close to the cervical cord.

**Figure 3:**
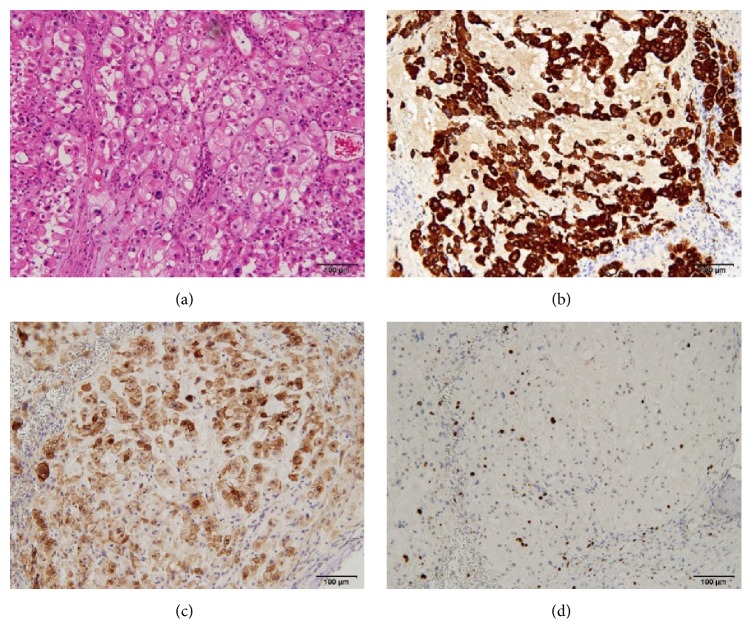
Histological and immunohistochemical features of the tumor. (a) Hematoxylin and eosin stain showing pathognomonic physaliphorous cells with multivacuolated cytoplasm. The appearance of chordoma consists of cords and islands of tumor cells arrayed in a distinctly lobular fashion, with mucus-filled cytoplasm. (b, c, d) The tumor cells demonstrating positive immunostaining with AE1/AE3 (b), epithelial membrane antigen (EMA) (c), and KI-67 expression (d). Scale bar of all the panels: 100 *μ*m.

The pathological diagnosis was chordoma. On pathological examination, the cytoplasm of the tumor cells was acidophil with considerable nuclear pleomorphism (Figure 3(a)). It was lobulated and composed of an abundant myxoid stroma, with distributed nests, sheets, and cords of univacuolated, multivacuolated (physaliphorous), and glandular cells that showed almost some degree of nuclear atypia (Figure 3(a)). Immunohistochemical analysis revealed that the tumor cells were positive for cytokeratin AE1/AE3 (Figure 3(b)). Epithelial membrane antigen (EMA: Figure 3(c)) and S-100 protein indicated a notochordal cell origin. The ratio of the MIB-1 index (Ki-67-positive cells) within the tumor was high (19.0%, Figure 3(d)).

Regarding the postoperative course, the patient underwent three additional spinal surgeries, because local recurrences of the initial lesion were found at 9 months, 4 years and 2 months, and 6 years after the first surgery (Figures 2(b) and 2(c)). All three recurrences were located in front of the dura and were clearly encapsulated and isolated as a small oval lesion. These recurring tumors were treated with en bloc resection through the anterior approach, followed by replacement of the titanium mesh cage (Figure 2(d)). At 8 years and 4 months after the first surgery, the patient was ambulatory without lower and upper limb paralysis and demonstrated no neurological symptoms without recurrence (Figures 2(e) and 2(f)).

## 3. Discussion

Generally, surgical resection with a wide margin is the most appropriate management for sacrococcygeal chordoma [16]. Tomita et al. [17, 18] described an innovative surgical technique, termed total en bloc spondylectomy for malignant vertebral tumors in the thoracolumbar spine. Total en bloc spondylectomy with a wide margin is generally accepted to be the most appropriate management for thoracic and lumbar lesions. However, this method is still technically challenging for the cervical spine because of the proximity of the tumor to the vertebral arteries and neural element. Several reports described experiences with total en bloc spondylectomy for cervical lesions. Fujita et al. [3] attempted en bloc resection for a C5 tumor with ligation of the left vertebral artery, resulting in tissue intralesional resection because the tumor had invaded the neural foramen. Rhines et al. [19] and Bailey et al. [20] reported the en bloc resection of a cervical chordoma that required sacrifice of the vertebral artery and nerve root. Currier et al. [21] reported a case of C5 chordoma treated successfully with total en bloc spondylectomy preserving the vertebral arteries and neural structures. The resection was performed with wide surgical margins because the tumor was localized inside the vertebral body without invading the foramen or spinal canal. They recommended total en bloc spondylectomy for cervical chordoma if the tumor is confined to the vertebral body [21].

The present case was treated with total piece meal spondylectomy, because the tumor invaded the neural foramen and spinal canal, compressing the neural element, which resulted in neurological symptoms. The three recurrences might be originated from the contamination of the tumor tissue even after thorough resection macroscopically with MRI confirmation. However, all the recurrences were fortunately located adjacent to the titanium mesh cage, encapsulated without adhesion to the dura. These conditions made the recurrent lesion easy to remove with additional surgery. The prognosis in cervical chordoma depends on not only the surgical procedures, such as total en bloc spondylectomy or total piecemeal spondylectomy, but also the histological features or age of the patient.

Microscopic tumor necrosis and MIB-1 index (Ki-67 level of >5%) are histologically associated with a significantly increased risk of local recurrence and metastasis, indicating the impact of certain factors on survival in case of chordoma [14]. The present patient had repeated local recurrence three times during the period of 8 years and 4 months after the initial surgery, and these instances were supported by histological findings of chordoma. Nevertheless, the 73-year-old patient survived after more than 8 years without metastasis, although the reported relative 5-year survival rate of patients with chordoma is 35% and the 10-year rate is 18%, and the incidence of local recurrence and metastasis was 30–60% in cases with long-term clinical follow-up data [22, 23]. Regardless of surgical procedures, Zhong et al. [24] reported that the survival rate and incidence of pulmonary metastasis were higher in the younger group (<25 years old) than in the senior group (>25 years old).

The rates of local recurrence and metastasis depend on the quality of the initial surgery, pathological features, and age of the patient. In the present case, tumor resection was performed via anterior and posterior approaches by total piecemeal spondylectomy but not en bloc resection, followed by three additional resections of the recurrent lesions. This resulted in successful spinal stabilization without neurological deficiency, and the patient was able to obtain good performance in activities of daily living without lower and upper limb paralysis, that is, good functional prognosis, at 8 years and 4 months after the initial surgery.

## 4. Conclusions

Piecemeal spondylectomy is an alternative management for cervical chordoma in aged patient even with high MIB-1 indexes.
